# Surgical management in submucous cleft palate patients

**DOI:** 10.1007/s00784-020-03719-1

**Published:** 2021-02-01

**Authors:** B. J. A. Smarius, C. H. A. L. Guillaume, J. Slegers, A. B. Mink van der Molen, C. C. Breugem

**Affiliations:** 1 Department of Pediatric Plastic Surgery, Wilhelmina Children’s Hospital, University Medical Center, Utrecht, 3508 AB Utrecht, The Netherlands; 2 Department of Plastic Surgery, Meander Medical Center, Amersfoort, The Netherlands; 3 Department of Pediatric Plastic Surgery, Emma Children’s Hospital, University Medical Center Amsterdam, Amsterdam, The Netherlands; 4 Department of Plastic Surgery, St. Antonius Hospital, Nieuwegein, The Netherlands

**Keywords:** Submucous cleft, Speech problems, Speech therapy, Otitis media

## Abstract

**Objectives:**

The submucous cleft palate (SMCP) is considered to be the most subtle type of cleft palate. Early detection is important to allow on time intervention by speech therapy and/or surgical repair before the children already develop compensatory speech mechanisms. The purpose of this study was to investigate at what time children with a SMCP present, to determine when children are operated, and to analyze the postoperative outcomes for in SMCP children.

**Patient and methods:**

Medical records from 766 individuals registered in the cleft registry in the Wilhelmina’s Children’s’ Hospital, Utrecht, were retrospectively reviewed. Inclusion criteria were children diagnosed with SMCP. The following data were collected: age at diagnosis, physical examination, age at surgery, surgical technique, speech therapy pre- and post-surgery, otitis media, secondary cleft surgery, family history, syndromes, and other anomalies.

**Results:**

In total, 56 SMCP children were identified. The mean age of diagnosis was 44.0 months (range 0–150, SD = 37.0). In 48 children (85.7%), surgical intervention was performed (Furlow plasty, intravelar veloplasty, pharyngoplasty, or Furlow combined with buccal flap).

**Conclusion:**

This retrospective study reconfirms that SMCP often presents late, even in a country with a modern healthcare system and adequate follow-up of all newborns by the so-called youth doctors in “children’s healthcare centers” up to the age of 4 years old. Almost 86% of patients ultimately needed palate surgery when SMCP was suspected.

**Clinical relevance:**

Any child presenting with repeated episodes of otitis media, nasal regurgitation, or speech difficulties should have prompt consideration for SMCP as diagnosis.

## Introduction

Cleft lip and/or palate (CLP) is one of the most prevalent congenital anomalies with a reported incidence of 13.5 in every 10,000 births and cleft palate only (CP) at 5.7: 10,000 live births [1]. The submucous cleft palate (SMCP) is often considered the most subtle type of all palate clefts. Prevalence of SMCP amongst children is reported between 0.02 and 0.08% [2–5].

SMCP is defined as an incomplete union of mesoderm, differentiating into muscle, across the soft palate, while the ectoderm does fuse, resulting in intact oral and nasal mucosa [6]. The SMCP can traditionally be characterized by the triad of a bifid uvula, zona pellucida, and a notch in the posterior surface of the hard palate, although not all patients will present with all three the characteristics of the submucous cleft palate [7].

Symptoms can vary depending on the age of the child. In young children, feeding difficulties and/or nasal regurgitation are most common [8, 9]. With progressing age, ear problems such as acute otitis media (AOM), otitis media with effusion (OME), and hearing problems may become more evident [10]. Speech difficulties associated with velopharyngeal insufficiency (VPI) occur in up to 80% of children with unrepaired SMCP [2, 3, 9]. Defective palatal muscles prevent adequate velopharyngeal closure during speech, and this results in speech characterized by increased nasal resonance (hypernasality), nasal air leakage, or turbulence [11]. Early detection is necessary to initiate speech therapy and to make it possible to operate early enough before patients develop compensatory speech mechanisms [9, 12].

CLP is almost always diagnosed in the first year of life, while CP is diagnosed only in 87.6% of children reviewed by Bell et al. before 12 months [13]. Hanny et al. demonstrated that 25% of all CP patients were diagnosed after 12 months of age [14]. SMCP is less visible and can therefore be easily missed during the first screening after birth and as a result of this is often diagnosed late [15]. Ten Dam et al. found that the diagnosis was made at a median age of 3.7 years [16]. Late detection can also occur due to the fact that a children start to speak complete sentences around 24 months of age making it easier to diagnose VPI [17].

The purpose of this study was to investigate at which point in time children with SMCP present, to determine when children are operated, and to analyze the postoperative speech outcomes in SMCP children.

## Patient and methods

### Clinical data

Child records from 766 individuals (all type of clefts) registered in the Dutch Association for Cleft Palate and Craniofacial Anomalies registry from 1997 to 2014 in the Wilhelmina’s Children’s’ Hospital, Utrecht, were retrospectively reviewed. Inclusion criteria were submucous cleft registration between 1992 and 2014, with children being at least 5 years of age.

Information regarding physical examination, age at diagnosis, age at primary palate surgery, surgical technique, speech therapy pre- and post-surgery, otitis media, secondary palate surgery, and syndromes/anomalies were extracted from their medical files (by BS and JS). Permission for this study was obtained from the Medical Ethics Review Committee (METC) Board at the University Medical Center, Utrecht, the Netherlands (reference number WAG/mb/18/038352).

### Physical examination

Physical examination was performed by the treating plastic surgeon. There were two indications to perform a physical examination. In the first group, oral examination was done before the age of 2.5 years for a suspected SMCP. All children had swallowing/feeding problems with either a notable abnormal or normal palate. In the second group, oral examination was done after the age of 2.5 years. In this group VPI triggered the diagnosis SMCP as hypernasal speech had developed.

All types were described according the triad of Calnan: bifid uvula, zona pellucida, and notched posterior border of the hard palate [7].

### Genetics

Like other cleft palate types, SMCP can occur as an isolated malformation or associated with a syndrome. The cleft team in Utrecht routinely offers the child and their parents a visit to the clinical genetics for counseling.

### Surgical intervention

Children who underwent surgery were categorized by operation type: intravelar veloplasty (IVV), Furlow Z-plasty, cranially based pharyngeal flap (pharyngoplasty), or a combined operation technique (Furlow combined with buccal flap). The IVV was performed during the von Langenbeck technique [18]. The Furlow’s technique was used as described by Furlow (1995) [19]. Pharyngoplasty was performed with cranial-based pharyngeal flap. Combined operations encompass a modified Z-plasty in combination with a buccal flap [20].

### Speech assessment

In the Wilhelmina Children’s Hospital, the speech therapists routinely assess the speech of all children with SMPC according the Dutch Cleft Speech Evaluation Test (DCSET) [21]. The speech therapists in Utrecht do participate in the national calibration sessions for the DCSET. The DCSET is performed at the minimum age of 2.5 years old. In children younger than 2.5 years old the, DECSET cannot be performed because they cannot pronounce sentences. All pre-operative and postoperative results (1 year after operation) of the DCSET were collected by the speech therapists.

Speech was assessed in the following order: resonance, nasal emissions, oral facial muscle function, intelligibility, articulation, and consonant production.

The resonance was subjectively evaluated while the child speaks loudly 6 nasal, 5 oronasal, and 6 oral sentences. Resonance was scored for each sentence on a 3-point scale. A score of 1 was given for normal resonance, and a score of 3 for severe hypernasality or hyponasality. Nasometry was only used in children > 4 years old. However, nasometry was not used to analyze the resonance in young children (< 4 years) because of the insufficient cooperation with the nasometer [22].

A mirror test was performed to detect nasal leakage. Orofacial function was observed during the assessment. Attention was paid to open mouth, tongue position, and mouth breathing.

The intelligibility was scored during spontaneous speech and was scored using a 5-point scale. A description of the intelligibility scores used by the parents and speech pathologists are presented in Table 1.

**Table 1:** Intelligibility score used by speech-language pathologist in the Wilhelmina

Children’s Hospital	
1	Always understandable for everybody without difficulty
2	Speech-disorder hearable, although understandable
3	Speech-disorder hearable, understandable with some difficulty
4	Speech-disorder hearable, understandable for family with some difficulty, however poorly understandable for strangers despite effort
5	Barely or not understandable for anyone despite effort

Finally, articulation was evaluated. Children were asked to speak words aloud and also sentences in a playful manner depending on the age of the child. If a misarticulation occurred, the type of error was indicated on the form.

### Acute otitis media (AOM) and otitis media with effusion (OME)

The presence or absence of AOM/OME was determined by physical examination and documented by the pediatric otolaryngologist. Also the insertion of ventilation tubes was recorded.

### Secondary surgery

If the speech assessment was satisfactory after primary surgery, the surgery was scored as successful. In case of unsatisfactory speech resulted and speech therapy did not help, secondary palate operation was performed to improve the speech.

### Complications

Complications were categorized as fistulas, bleeding, infection, delayed wound healing defined as requiring more than 2 weeks of anticipated healing or involving superficial ulceration, and wound dehiscence.

### Statistics

SPSS statistics version 25 for windows (SPSS Inc., Chicago, Il, USA) was used for statistical analysis. The Fisher’s exact test was used for associations between categorical variables. The Wilcoxon signed rank test was used for data analysis of the intelligibility scores because the repeated measurements on a single sample. Significance for differences was expressed using *p* values. A *p* value of < 0.05 was considered to be significant.

## Results

### Patient characteristics

In total, 56 SMCP children were diagnosed. SMCP children constituted 7.3% (56/766) of the cleft population in the Wilhelmina Children Hospital. Fifty percent (*n* = 28) of the children were boys. The mean age at diagnosis was 44.0 months (range 0–150, SD = 37.0). Family history of clefts was documented in seven child records (13.2%). All characteristics are listed in Table 2.

**Table 2:** Patient characteristics

*Characteristics*	*Patients* *n = 56 (%)*
***Gender***	
Male	28 (50)
Female	28(50)
***Age***	
Mean age at diagnosis	44.0 months (range 0–150, SD 37)
Mean age at operation	53.4 months (range 4–160, SD 35.9)
Age < 2.5 years old	13
Age > 2.5 years old (included not operated children)	43
***Submucous cleft characteristics***	
No signs of submucous cleft	7 (12.5)
Bifide uvula	11 (19.6)
Zona pellucida	1 (1.8)
Notch hard palate	0 (0)
Bifide uvula + zona pellucida	35 (62.5)
Bifide uvula + zona pellucida + notch hard palate	2 (3.6)
***Operation***	
No	8 (14.3)
Yes	48 (85.7)
***Operation technique***	
Intravelar veloplasty	30
Furlow Z-plasty	4
Cranial based pharnyngeal flap	11
Combination (IVV with buccal flap)	3
***Ear problems***	
History of AOM/ OME	35 (62.5)
Tubes placed	21(37.5)
Positive cleft family history	7 (12.5)

### Syndromes and other anomalies

Of the 56 children with SMCP, 31 (55.4%) parents consented to be referred to the department of clinical genetics for evaluation and possible testing. In total 32.1% of the children (18/56) was diagnosed with a syndrome and 19.6% of the children (11/56) had other anomalies. Velocardiofacial syndrome (VCF) was the most common syndrome (*n* = 4). All syndromes and anomalies are listed in Table 3.

**Table 3:** Syndromes and other anomalies

**Patients overall**	*56 (100)*
Clinical genetic test	
Yes	*31 (55.4)*
No	*25 (44.6)*
Syndrome overall	
Yes	*18 (32.1)*
No (confirmed by test)	*13 (23.2)*
Other anomalies overall	*11 (19.6)*
**Subgroup: patients without operation**	*8 (14.3)*
Clinical genetic test	
Yes	*6*
No	*2*
Syndrome	*4*
Stickler syndrome	*2*
Kabuki syndrome	*1*
VCF syndrome	*1*
Other anomalies	*2*
Microtia	*1*
Hemifacial microsomia	*1*
**Subgroup: patients with operation**	*48 (85.7)*
Clinical genetic test	
Yes	*25*
No	*23*
Syndrome	*14*
VCF syndrome	*4*
Stickler syndrome	*1*
Apert syndrome	*1*
Kabuki syndrome	*1*
Down syndrome	*1*
KBG syndrome	*1*
Charge syndrome	*1*
Loeys-Dietz syndrome	*1*
DOOR syndrome	*1*
18q syndrome	*1*
Auriculo-condylar syndrome	*1*
Other anomalies	*9*
Psychomotor retardation	*3*
Plagiocephaly; psychomotor retardation	*1*
Microtia	*1*
Hemifacial microsomia with microtia	*1*
Pierre Robin Sequence	*1*
Trigonocephaly	*1*
Microcephaly and hydrocephalus	*1*

### Physical oral examination

The type of SMCP was initially determined during physical examination. All types were described according the triad of Calnan: bifid uvula, zona pellucida, and notched posterior border of the hard palate. Physical oral examinations are listed in Table 2.

### Surgical intervention

In 85.7% of the children (48/56), surgical intervention was performed. The mean age at time of operation for all children was 53.4 months (range 4–160, SD 35.9). Comparing the group younger than 2.5 years of age with the group older than 2.5 years, mean ages at operation were 18.4 months respectively (range 4–24, SD9.0) and 66.4 months (range 31–160, SD33.3). In 62.5% of the children (30/48), an IVV was performed, in 22.9% of the children (11/48) a cranial based pharyngeal flap, in 8.3% of the children (4/48) a Furlow plasty, and in 6.3% of the children (3/48) a combined operation (Furlow combined with a buccal flap). Eight children (14.3%) did not require surgical correction as they did not develop VPI. Six of the 8 children had a normal resonance and two children improved to satisfactory results with just speech therapy. Physical oral examination of these children showed in four a bifid uvula only and in the other four a bifid uvula with zona pellucida.

### Speech assessment

For speech analyses, data from 48 children were available for pre- and/or post-surgery DCSET. Of the 48 children 13 children were younger than 2.5 years at time of operation and did not get a preoperative DCSET because this was not possible at this young age. Preoperative DCSET data were missing from 3 children and postoperative DCSET data were missing from 1 child. After these exclusions preoperative data from 32 children and postoperative data from 47 children were available. The mean time of postoperative DCSET was 14.51 months (range 4–57, SD 9.5) after surgery. Table 4 shows the results of all DCSETS in children who required surgery.

**Table 4:** Pre- and postoperative DCSET scores, AOM/OME and syndromes/anomalies for all patients who underwent an operation

	**Gender**	**Operation type**	**DCSET preoperative**	*Resonance*	*Nasometry*	*Nasal emission*	*Oral facial muscle function*	*intelligibility speech therapist*	*intelligibility parents*	*Articulation problem*	**DCSET postoperative**	*Resonance*	*Nasal emission*	*Oral facial muscle function*	*intelligibility speech therapist*	*intelligibility parents*	*Articulation problem*	**Secundary operation**	**AOM/ OME**	**Syndrome/ Anomalie**
**<2.5 Years old**																				
1	M	IVV	No	N/A	N/A	N/A	N/A	N/A	N/A	N/A	Yes	1	–	–	2	2	–	No	–	Stickler syndrome
2	M	IVV	No	N/A	N/A	N/A	N/A	N/A	N/A	N/A	Yes	1	–	–	1	1	–	No	+	No syndrome
3	F	Furlow	No	N/A	N/A	N/A	N/A	N/A	N/A	N/A	Yes	3	+	–	3	2	–	No	+	Charge syndrome
4	F	IVV	No	N/A	N/A	N/A	N/A	N/A	N/A	N/A	Yes	1	–	+	1	1	–	No	+	DOOR syndrome
5	F	IVV	No	N/A	N/A	N/A	N/A	N/A	N/A	N/A	Yes	3	+	–	4	4	–	Yes	+	No syndrome
6	M	IVV	No	N/A	N/A	N/A	N/A	N/A	N/A	N/A	Yes	2	+	+	3–4	3	+	Yes	+	Auricula-condylar-Syndrome
7	F	IVV	No	N/A	N/A	N/A	N/A	N/A	N/A	N/A	Yes	1	–	–	2	2	–	No	–	Loeys-Dietz-Syndrome
8	F	IVV	No	N/A	N/A	N/A	N/A	N/A	N/A	N/A	Yes	1	–	–	1	1	–	No	+	Not tested
9	F	IVV	No	N/A	N/A	N/A	N/A	N/A	N/A	N/A	Yes	1	–	–	2	1–2	+	No	+	No syndrome; Psychomotore retardation
10	M	IVV	No	N/A	N/A	N/A	N/A	N/A	N/A	N/A	Yes	1	–	–	1	1	–	No	+	Not tested
11	M	IVV	No	N/A	N/A	N/A	N/A	N/A	N/A	N/A	No	N/A	N/A	N/A	N/A	N/A	N/A	No	–	Not tested
12	F	IVV	No	N/A	N/A	N/A	N/A	N/A	N/A	N/A	Yes	1	–	–	1–2	1	–	No	+	Not tested
13	F	IVV	No	N/A	N/A	N/A	N/A	N/A	N/A	N/A	Yes	2	+	–	4	4	+	No	+	No syndrome; Pierre Robin sequence
**>2.5 Years old**																				
14	F	Furlow + buccal flap	Yes	3	–	+	+	3	3	+	Yes	1	–	–	2	2	–	No	–	Not tested
15	M	IVV	No	N/A	N/A	N/A	N/A	N/A	N/A	N/A	Yes	1	–	–	2–3	2	+	No	+	No syndrome
16	F	Furlow	Yes	2	–	+	+	2	3	+	Yes	1	–	–	1–2	1–2	–	No	–	Down syndrome
17	M	Furlow	Yes	2	–	+	–	3	2–3	–	Yes	1	–	–	1–2	2	–	No	–	No syndrome
18	M	Furlow + buccal flap	Yes	3	+	+	+	2–3	2–3	+	Yes	1	–	–	2	1	–	No	+	Not tested
19	M	Pharyngoplasty	Yes	3	+	+	+	4	4	+	Yes	2	–	–	2	2	–	No	+	Not tested
20	M	IVV	Yes	2	+	+	+	2	2	–	Yes	1	–	–	1	2–3	–	No	+	Not tested
21	F	IVV	Yes	3	–	+	–	4	4	–	Yes	3	+	+	3	3–4	–	Yes	+	No syndrome
22	F	IVV	Yes	3	–	+	+	5	5	+	Yes	3	+	+	5	5	+	Yes	+	No syndrome; microcephaly and hydrocephalus
23	M	IVV	Yes	3	+	+	+	3	3	–	Yes	2	+	+	2	3	–	Yes	–	No syndrome
24	F	IVV	Yes	3	+	+	–	3	3	–	Yes	2	+	–	2	2	–	Yes	–	KBG syndrome
25	M	IVV	Yes	3	–	+	–	3	3	–	Yes	3	+	–	3	3	–	Yes	–	VCF
26	M	IVV	Yes	3	–	+	+	4	4	+	Yes	1	–	–	1	1	–	No	+	Not tested; Trigonocephaly
27	F	IVV	No	N/A	N/A	N/A	N/A	N/A	N/A	N/A	Yes	3	+	–	3	3	–	Yes	+	Not tested; Plagiocephaly; psychomotore retardation
28	F	Pharyngoplasty	Yes	3	–	+	–	4	4	+	Yes	1	–	–	1–2	1–2	–	No	–	Not tested
29	M	IVV	Yes	3	–	+	–	5	5	+	Yes	3	+	–	4	4	+	Yes	–	Not tested
30	M	IVV	Yes	3	+	+	–	3	3	+	Yes	3	+	–	3	3	+	Yes	–	VCF
31	F	Pharyngoplasty	Yes	3	+	+	–	3–4	3–4	–	Yes	1	–	–	1	1	–	No	+	Not tested
32	F	IVV	Yes	2	–	+	–	2	2	–	Yes	1	–	–	1	1	–	No	+	Not tested
33	F	IVV	Yes	3	+	+	–	3	3	–	Yes	1	–	–	1	1	–	No	+	No syndrome
34	M	Pharyngoplasty	No	N/A	N/A	N/A	N/A	N/A	N/A	N/A	Yes	1	–	–	1	1	+	No	+	Not tested; psychomotor retardation
35	F	Pharyngoplasty	Yes	3	–	+	–	4–5	4–5	+	Yes	1	+	+	3	2–3	+	No	+	Kabuki syndrome
36	M	IVV	Yes	3	+	+	–	3	3	–	Yes	1	–	–	2	2	–	No	+	Apert syndrome
37	M	IVV	Yes	2	–	+	+	3–4	3–4	–	Yes	1	–	–	2	2–3	–	No	+	Not tested; hemifacial microsomia with microtia
38	F	Pharyngoplasty	Yes	3	+	+	+	3	3	–	Yes	1	–	–	2	2	–	No	+	Not tested
39	M	IVV	Yes	3	–	+	+	5	5	+	Yes	3	+	+	5	5	+	Yes	+	18q syndrome
40	F	IVV	Yes	3	–	+	–	4	4	+	Yes	2	+	–	3	3	–	Yes	+	Not tested
41	F	Furlow	Yes	3	–	+	+	4	4–5	+	Yes	3	–	+	3	3–4	–	Yes	+	VCF
42	M	Furlow + buccal flap	Yes	2	–	+	+	3	3	–	Yes	2	+	–	2–3	2–3	–	Yes	–	No syndrome; Mircrotia
43	F	Pharyngoplasty	Yes	3	+	+	+	5	4–5	+	Yes	2	+	+	4	3–4	+	No	+	No syndrome; psychomotor retardatin
44	F	Pharyngoplasty	Yes	3	–	–	+	4	3	+	Yes	1	–	–	2	2	+	No	+	VCF
45	m	IVV	Yes	3	+	+	+	2–3	2–3	–	Yes	3	+	+	3–4	3–4	–	Yes	+	Not tested
46	M	Pharyngoplasty	Yes	3	+	+	–	2–3	2–3	+	Yes	1	–	–	2	2	–	No	–	Not tested
47	M	Pharyngoplasty	Yes	3	–	+	–	5	5	+	Yes	1	–	–	1	1	–	No	+	Not tested
48	M	Pharyngoplasty	Yes	3	+	+	+	5	4	+	Yes	2	–	+	3	3–4	–	No	–	No syndrome

AOM/OME: Acute otitis media/ Otitis media with effusion; IVV: Intravelar veloplasty; KBG syndrome: The name of the syndrome is based on the initials of the first 3 patients reported by Hermann et al. in (1975); VCF: Velocardiofacial syndrome

### Resonance

Hypernasality was scored perceptually and in some cases also a preoperative nasometry was performed. Preoperative 81.3% of the children (26/32) had severe hypernasality and 18.8% of the children (16/32) had light to moderate hypernasality. Postoperative 23.4% of the children (11/47) had still severe hypernasality, 19.1% of the children (9/47) had light to moderate hypernasality and 57.4% of the children (27/47) had normal perceptual resonance. Nasometric scores were obtained for 14 of the children. All children showed increased nasalance (> 2SD) scores compared with the normal values.

### Nasal emission

Preoperative in 96.9% of the children (31/32) there was nasal emission using the mirror test. The postoperative mirror test showed nasal emission in 38.3% of the children (18/47).

### Orofacial muscle function

Special attention was given to abnormal open mouth, tongue position, or mouth breathing. Preoperative in 53.1% of the children (17/32) an abnormal orofacial muscle function was observed. Postoperatively this number decreased to 23.4% of the children (11/47).

### Intelligibility of speech

The intelligibility scores preoperative en postoperative evaluated by the speech pathologist and the parents are presented in Table 4. The mean level of preoperative intelligibility (only children > 2.5 years old) was 3.5 (range 2–5) and 3.5 (range 2–5) as evaluated by the speech pathologist and parents, respectively. The mean level of postoperative intelligibility was 2.3 (range 1–5) and 2.4 (range 1–5) as evaluated by the speech pathologist and parents, respectively. There was a significant (*p* = < 0.001 speech therapist, *p* = < 0.001 parents) intelligibility improvement after surgery.

Some children had still unsatisfactory speech after operation (Table 4). Fifteen chose for a secondary surgery (see secondary surgery). In the other cases parents did not opt ​​for reoperation because of other problems.

### Articulation

The presence of preoperative articulation errors was documented in 56% of the children (18/32). Postoperative articulation errors were found in 25.5% of the children (12/47).

### Acute otitis media (AOM) and otitis media with effusion (OME)

Almost 63% of the children (35/56) reported an onset of AOM/OME. Thirty-eight percent of the children (21/56) underwent insertion of ventilation tubes because of consequent conductive hearing loss.

### Secondary surgery

Secondary surgery was performed in 31.3% of the children (15/48) for unsatisfactory speech assessment scores. Fourteen percent of these children (6/15) were known with a syndrome. There was no significant difference (*p* = 0.528) in secondary surgery between syndrome and non-syndrome. Two of the 15 children were operated before the age of 2.5 years, and 13 of them were operated after the age of 2.5 years. There was no significant difference (*p* = 0.182) in secondary surgery between the two groups (< 2.5 years vs. > 2.5 years). After secondary surgery, 12 of the 15 children had satisfactory speech assessment scores and 3 did not. In these last three children, the parents did not choose ​​for a reoperation because of other (extensive) problems.

### Complications

In the 48 operated SMCP children, 2 (4.2%) complications occurred. One (2.1%) child developed a postoperative fistula. The parents of this child decided to be treated in another hospital. There was 1 (2.1%) child with postoperative bleeding 2 days after operation and went to the operation room to obtain hemostasis.

## Discussion

SMCP is less visible and can be easily missed during the after birth screening. Early detection is mandatory to initiate speech therapy on time and to make it possible to have an early surgery compensatory speech mechanisms develop.

The present study found in 7.3% of all cleft patients in clinical setting to have SMCP, which corresponds with the findings of Crikelair et al. (1970) who found SMCP in 4% of all cleft patients [23]. Primary palate surgery was performed in 85.7% of the SMCP children.

The present study showed that children with a submucous cleft routinely present late (44.0 months, range 0–150, SD = 37.0). This is just a bit earlier than in the study of Reiter et al. who found a mean age of diagnosis at 4.9 years and comparable with Brosch et al. who found a mean age at 4.2 years [24, 25]. In the present study and Reiter’s study, there is a very wide range, meaning that SMCP is not always noted early in life.

The mean age at time of operation in the present study was 53.4 months (4.5 years). This corresponds with the literature (range 3.9–7.7 years) [9, 26–28]. There was delay of almost 1 year been age at diagnosis (44 months) and age at surgery (53.4 months). All children were treated with speech therapy first to improve the speech. However, the exact duration of speech therapy was unknown. Another conceivable explanation for this 1 year delay could be the operation waiting list. Unfortunately, this could not be distinguish from the data.

The present study reports syndromes in 32.1% cases, which is comparable with Sullivan et al. (2011), who found 28% to have syndromes [29]. However, Reiter (2011) found a lower percentage of 17.9% [24]. Syndromes might be an additional cause for persisting problems or limited success of additional speech therapy needed in moderate or severe cases after intervention. For example, patients with Down syndrome score lower on measures of phonological accuracy and occurrence of phonological processes [30]. However, in the present study, there was no significant difference in secondary surgery between syndrome and non-syndrome children.

The triad of Calnan is accurately described in the literature [7]. A bifid uvula was present in 59–98% of the SMCP patients, notched posterior border of the hard palate in 68.1–100%, a zona pellucida in 45.1–85%, and occult SMCP in 25% [4, 9, 24, 29, 31]. Not every SMCP patient showed the typical Calnad triad. In the present study, a bifid uvula was noted in 85.7% of the patients (48/56), which corresponds with previous literature. All three sights (bifid uvula, bony notch and zona pellucida) were only described in 3.6% of the patients (2/56). In Chen et al. (1994), the three symptoms together were also not investigated [32]. The lack of awareness of SMC and its variability in presentation may be the reason that it often goes undetected [24]. In the Netherlands, the follow-up of all newborns is handled by “children’s healthcare center” up to the age of 4 years. In any child presenting with repeated episodes of otitis media, nasal regurgitation, or speech difficulties, the diagnosis SMCP should be considered. Awareness of health professionals working at the “children’s healthcare center” about possible underlying SMCP at the children’s healthcare is of great importance for the early diagnosis. More awareness can be achieved through education about symptoms of SMCP and approachable if health professionals have any doubt.

Surgical intervention for SMCP is hampered by a lack of good clinical comparative studies. The discussion focuses on restoring velopharyngeal competence, which theoretically can be done by restoring the palatine muscles in a more dorsal position with or without velum lengthening, using a pharyngeal flap, or a combination [29, 33]. However, since some patients with SMCP develop normal speech, there is an ongoing debate whether to operate early or only when speech problems become apparent.

Recent years more studies are published mentioning positive operation outcomes using Furlow-plasty in SMCP children [27, 29, 31, 34, 35]. Sullivan et al. (2010) recommend double-opposing Z-palatoplasty as the primary operation for children younger than 4 to 5 years with SMCP (overt or occult) and velopharyngeal insufficiency [29]. Chen et al. (1996) and Seagle et al. (1999) reported satisfactory success rates of 96.7 and 96%, respectively, using Furlow-plasty by SMCP [27, 31]. Unfortunately, due to many different intervention techniques of the present study, numbers are too small to draw conclusions. Another additional problem is the difference in outcome measurement in each study making comparison between studies cumbersome.

Due to screening a prevalence of OME ranging from 15–40% was found in the general population with incidences in the cleft population up to 97% [34, 36, 37]. Previous studies on OME in the SMCP population found prevalence’s of 49% [9]. The present study found the prevalence of AOM/OME to be 62.5%, which is high compared with the general population. Due to the anatomical abnormalities (dysfunction of the tensor veli palatine muscle) present in SMCP, it is reasonable that a large number of SMCP patients would suffer from concomitant Eustachian tube dysfunction [38].

This study has several limitations. Due to its retrospective nature, there are some inherent weaknesses; however comparisons to the published literature can be made. The small numbers of patients per operation type in this study make it difficult to compare between operation types. The study should be classified as an observational study and not as a study to comparing different operation techniques.

There is an interesting review about the management of SMCP from Gilleard et al. This study found little evidence to support any specific surgical intervention due to the mixed etiologies within the study population and the lack of unbiased validated preoperative and postoperative speech assessment [39]. To recommend for a secure evidence-based surgical management of SMCP, further methodologically rigorous studies are needed.

## Conclusion

This retrospective study reconfirms that SMCP often present late (mean 44 months) and that almost 86% patients ultimately need palate surgery when SMCP is suspected. Parents should be informed about these findings prior speech therapy. Therefore, in children presenting with repeated episodes of otitis media, nasal regurgitation, or speech difficulties, the diagnosis of SMCP should be considered.
